# Children living in institutional care: How can mentalization-based interventions improve their perspective-taking and conflict resolution skills?

**DOI:** 10.1192/j.eurpsy.2022.1772

**Published:** 2022-09-01

**Authors:** B. Szabó, E. Nagy, A. Békefi, J. Futó

**Affiliations:** Eötvös Loránd University, Department Of Developmental And Clinical Child Psychology, Budapest, Hungary

**Keywords:** intervention, institutional care, Children, mentalization

## Abstract

**Introduction:**

Trauma, stress, and attachment problems are negatively related to the development of mentalization. Children raised in institutional care are more exposed to these difficulties, therefore the development of population-specific interventions that aim to improve mentalization skills would be highly desirable.

**Objectives:**

Our goal is to develop mentalization-based intervention programs for specific age groups (9-13 years, 14-18 years, and adult staff members of institutional care centers) - that support children’s and adolescents’ social functioning and conflict resolution skills.

**Methods:**

The mentalization-based intervention targeting institutional care staff was launched first. Due to the pandemic, this intervention was executed online with two intervention (*N* = 17) and two passive control (*N* = 15) groups. Before and after the intervention, participants completed a demographic questionnaire, the Parenting Sense of Competence Scale, the Reflective Functioning Questionnaire, the Mini Oldenburg Burnout Inventory, The Strengths, and Difficulties Questionnaire, and the Ways of Coping Questionnaire.

**Results:**

The intervention protocol and our results will be shown at the conference. There was no significant difference between the two intervention and two passive control groups in the demographic features. Mentalization uncertainty and burnout was positively related(*r*_s_(23) = .42, *p* = .034), while mentalization uncertainty and parental competence was negatively associated (*r*_s_(23) = - .41, *p* = .041).

**Conclusions:**

The intervention program will be fine-tuned and optimized based on the results of the pilot study. In the next interventions, we plan to focus on the issues that the staff perceived as most difficult and to conduct interventions among the children.

**Disclosure:**

No significant relationships.

